# Evaluation of mental stress by physiological indices derived from finger plethysmography

**DOI:** 10.1186/1880-6805-32-17

**Published:** 2013-10-12

**Authors:** Emiko Minakuchi, Eriko Ohnishi, Junji Ohnishi, Shigeko Sakamoto, Miyo Hori, Miwa Motomura, Junichi Hoshino, Kazuo Murakami, Takayasu Kawaguchi

**Affiliations:** 1 Graduate School of Comprehensive Human Sciences, University of Tsukuba, 1-1-1 Tennodai, Tsukuba, Ibaraki 305-8577, Japan; 2 Bio-Laboratory, Foundation for Advancement of International Science, 3-24-16 Kasuga, Tsukuba, Ibaraki 305-0821, Japan; 3 Department of Nursing, Ibaraki Prefectural University of Health Sciences, 4669-2 Ami, Inashiki, Ibaraki 300-0394, Japan; 4 Department of Intelligent Interaction Technologies, University of Tsukuba, 1-1-1 Tennodai, Tsukuba, Ibaraki 305-8577, Japan; 5 Faculty of Medicine, University of Tsukuba, 1-1-1 Tennodai, Tsukuba, Ibaraki 305-8577, Japan; 6 Department of Food and Nutrition, Faculty of Home Economics, Tokyo Kasei University, 1-18-1 Kaga, Itabashi, Tokyo 173-0003, Japan

**Keywords:** Chaos analysis, Color-word conflict test, Finger plethysmography, Mental stress, Peripheral circulation

## Abstract

**Background:**

Quantitative evaluation of mental stress is important to prevent stress-related disorders. Finger plethysmography (FPG) is a simple noninvasive method to monitor peripheral circulation, and provides many physiological indices. Our purpose is to investigate how FPG-derived indices reflect on mental stress, and to clarify any association between these physiological indices and subjective indices of mental stress.

**Methods:**

Thirty-one healthy women (mean age, 22 years ± 2) participated. The participants rested by sitting on a chair for 10 min. They then performed a computerized version of the Stroop color-word conflict test (CWT) for 10 min. Finally, they rested for 10 min. FPG was recorded throughout the experiment. The participants completed a brief form of the Profile of Mood States (POMS) questionnaire before and after the test. Using the FPG data, we conducted chaos analysis and fast Fourier transform analysis, and calculated chaotic attractors, the largest Lyapunov exponent, a high-frequency (HF) component, a low-to-high-frequency (LF/HF) ratio, finger pulse rate and finger pulse wave amplitude.

**Results:**

The HF component decreased and the LF/HF ratio increased significantly during the test (*P* < 0.01), while the confusion subscale of POMS increased after the test (*P* < 0.05). During testing, finger pulse rate significantly increased (*P* < 0.001), and the finger pulse wave amplitude decreased (*P* < 0.001). The attractor size reduced during testing and returned to a baseline level afterwards. Although the largest Lyapunov exponent showed no significant change during testing, significant negative correlation with the tension-anxiety subscale of POMS was observed at the beginning (*P* < 0.01). A significant negative correlation between the LF/HF ratio and two subscales was also observed in the beginning and middle of the test (*P* < 0.05). There were no correlations during the rest periods.

**Conclusions:**

The physiological indices derived from FPG were changed by mental stress. Our findings indicate that FPG is one of the easiest methods to evaluate mental stress quantitatively. In particular, the largest Lyapunov exponent and the LF/HF ratio might be associated with acute mental stress. Farther examination is needed to find any association between the physiological indices and various types of mental stress.

## Introduction

In recent years, the prevalence of depression and other stress-related illnesses has increased [1], and mental stress or job stress has been considered a risk factor for various illnesses [2-4]. High levels of mental stress can clearly contribute to cardiovascular disease, such as coronary artery disease and myocardial ischemia [5-8]. Identification of an individual’s stress level is the first step in stress studies for primary prevention of stress-related diseases. Although several studies have measured mental stress using such biomarkers as salivary cortisol and salivary alpha-amylase [9,10], these studies are expensive and require special expertise. Quantitative assessment of mental stress in a low-cost, noninvasive manner to obtain rapid results is thus regarded as an important goal.

Finger plethysmography (FPG) is a simple, noninvasive, well-known method for monitoring peripheral circulation [11]. There are commercially available devices to measure FPG, for example Finometer (Finapres Medical Systems, Amsterdam, Netherlands). Peripheral blood vessels contain a high concentration of arteriovenous anastomosis, innervated by alpha-adrenergic nerve fibers [6]. Peripheral blood flow thus reflects autonomic nervous system activity, which is commonly known as one indicator of mental stress. Although indices of autonomic nervous system activity are usually calculated using heart rate variability (HRV) [12-14], a number of recent reports have noted that finger pulse rate variability has nearly the same physiological function as HRV [15-17]. Because measurement of HRV usually requires electrodes to be attached to the chest or stomach, and electrodes sometime pick up noise from body movements, FPG is a superior method of measuring acute mental stress. Measuring FPG only requires putting the finger in a device. It is a minimum burden on users, and it can accurately measure changes in peripheral blood flow. Furthermore, it has been proposed that the FPG waveform reflects health conditions, with the signal becoming simpler and weaker as a result of disease or aging [18-20]. Goor *et al*. [21] demonstrated that peripheral arterial vasoconstriction induced by mental stress predicts stress-induced myocardial ischemia. They described that acute mental stress will lead to sympathetic nervous system activation and consequent peripheral vasoconstriction. Chronic stress may lead to peripheral blood ischemia and, consequently, cardiovascular disease (that is, coronary artery disease). Thus, measuring FPG during stress is important as a mean of predicting health outcome.

To quantify nonlinear signals, such as the pulse wave, we used nonlinear time-series analysis. Nonlinear dynamic analysis is an effective way of extracting regularity hidden in the peripheral circulation. Chaos analysis is a type of nonlinear time-series analysis and is an important part of mathematical models of complex systems science [20]. Chaos analysis enables simple regularity to be obtained from seemingly random data. One of the indices of chaos is a chaotic attractor [22]. An attractor is defined as simply a state of a dynamic system in the present study. Tsuda *et al*. [22] constructed a chaotic attractor from fingertip capillary vessels, identifying chaotic pulsation in these exponents. They reported that the shape of the chaotic attractor visualizes chaos statements, and reflects the age and health conditions of an individual. In addition, another index of chaos status, called the largest Lyapunov exponent, quantitatively expresses the degree of instability in orbital change of the chaotic attractor. The advantage of this assessment of peripheral dynamics is its high sensitivity. Imanishi and Oyama [23] suggested that the largest Lyapunov exponent of FPG was a more sensitive physiological index than the indices of autonomic nervous system activity. Mayumi and Miao [24] also reported that the Lyapunov exponent decreased in older people with severe dementia. We therefore surmised that the indices derived from these two types of analysis sensitivity would be associated with an extensive range of stress and that measuring FPG can be used to evaluating mental stress quantitatively.

However, few studies clarify the association between physiological indices derived from FPG and subjective indices of mental stress. The aim of this study was to investigate how physiological indices derived from FPG reflect acute mental stress and to figure out which index responds to which type of emotional change.

## Methods

### Study population

Thirty-one healthy women (mean age, 22 ± 2 years) participated in this study. We recruited participants by posting announcements on university bulletin boards. Eligibility criteria were: no underlying disease, especially no positive history of cardiovascular disease such as heart disease; no medication being taken; no color blindness; nonsmoker; not pregnant; and no prior experience of CWT. Participants were asked to avoid the following: a lack of sleep; intake of caffeine for at least 12 h before the experiment; excessive alcohol consumption; activities causing extreme physical fatigue before the experiment; and eating for 2 h before the experiment. The present study was approved by the research ethics committee of the Graduate School of Human Sciences, University of Tsukuba on September 2, 2010 (certification no. 22–179). Participation was voluntary and all participants provided written informed consent.

### Study design

**Figure 1:**
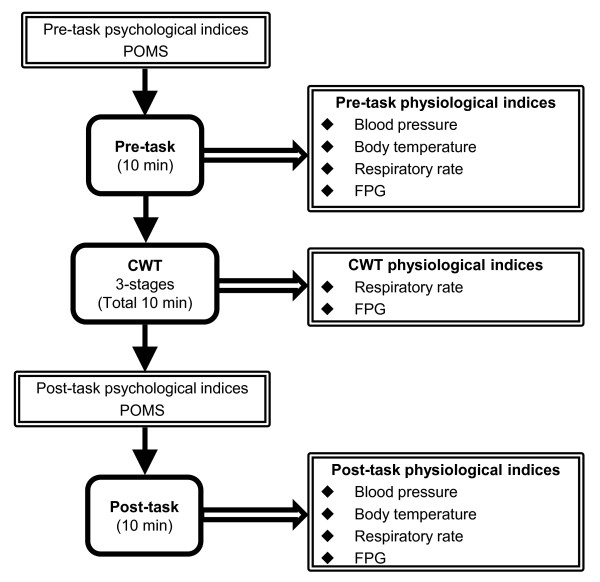
**Summary of experimental procedure.** CWT, Stroop color-word conflict test; FPG, finger plethysmography; POMS, Profile of Mood States; post-task, resting period after test; pre-task, resting period before test.

We used a before-and-after study design. The experimental procedure is shown in Figure 1. First, the mood of participants immediately before the experiment was evaluated using the Japanese version of the brief form of the Profile of Mood States (POMS) questionnaire, as a subjective psychological index. The POMS questionnaire covers six scales with 30 questions each: tension-anxiety; depression-dejection; anger-hostility; vigor; fatigue; and confusion [25]. Internal consistency for the POMS questionnaire, estimated by Cronbach’s alpha statistic, is 0.78. Next, participants remained quietly seated in a chair for 10 min. This rest period was designated 'pre-task’; during this time, the following physiological indices were measured: blood pressure; body temperature; respiratory rate; and FPG. Participants then performed the CWT in three stages of difficulty for a total of 10 min, during which FPG and respiratory rate were measured. Immediately after the CWT, POMS was again applied to assess mood (Cronbach’s alpha statistic, 0.73). Finally, participants were asked to rest for another 10-minute period, designated 'post-task’, during which physiological indices were again measured.

### Acute mental stress test

The CWT is a validated and widely used test that was proposed by Stroop [26] and is known to induce mental stress. Hoshikawa and Yamamoto [27] and Takei, Hama, and Yanagida [28] observed significant increases in heart rate during the CWT. Based on these protocols, we devised an original program in which tasks were performed on a computer screen, and the participant responded with mouse clicks. The tasks used the *kanji* characters for red, blue, green, yellow, and purple, and the correct color had to be selected from five colors presented randomly at the bottom of the screen. The test had three levels of difficulty, made by shortening the duration for which each task was presented. The time for a single task to be presented was 2.0 s in stage 1 (CWT-1), 1.6 s in stage 2 (CWT-2), and 1.4 s in stage 3 (CWT-3). Each stage lasted 3 min, and stages were presented consecutively for a total test time of 10 min.

### Measurement of physiological indices

Blood pressure was measured using a noninvasive sphygmomanometer (BP-608 Evolution II CS; Colin Medical Technology, Komaki, Japan), body temperature was measured with an ear-use thermometer (MC-510; Omron Healthcare, Kyoto, Japan), and respiratory rate was determined by watching participant’s shoulders and chest for 1 min. A skilled registered nurse in the research team measured the respiratory rate accurately. Because some participants may consciously change their respiration, we did not use an electronic strain gauge or other sensor. The FPG was measured using a pressurized pulse wave-detecting device that we developed in conjunction with CCI (Hakata, Japan).

### Measurement of FPG

**Figure 2:**
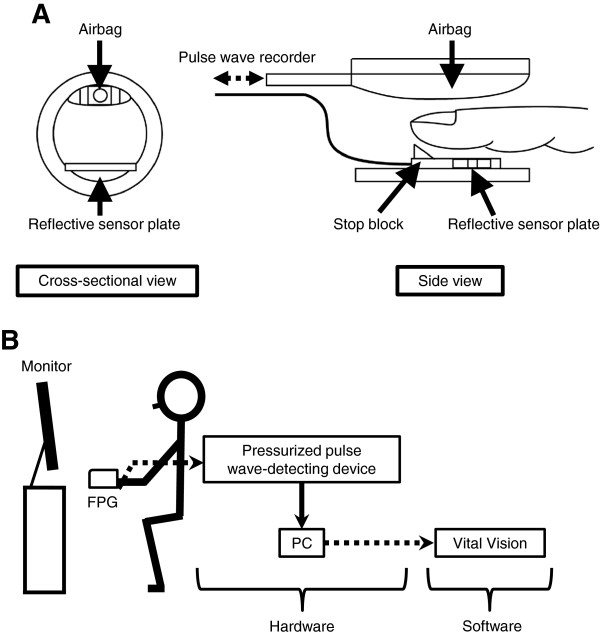
**Finger plethysmography. (A)** Pressure-controlled cuff with reflective sensor plate in finger plethysmography. Air pressure in the airbag at the top is adjusted by a pressure-controlled pulse wave recorder so that the fingertip is held lightly in place. The reflective sensor below the finger detects the finger pulse wave from the surface of the skin. The stop block acts as a guide to keep the finger in the correct position. **(B)** Schematic of measurement and analysis systems of finger plethysmography. FPG, finger plethysmograph; PC, personal computer.

To construct a chaotic attractor, we measured FPG using the instrument that we developed. A schematic diagram of the instrument is shown in Figure 2A. The finger was covered by an airbag and subjected to a slight cuff pressure of 20 mmHg to ensure stable contact between the skin surface and the sensor. At the same time, covering the fingertip with the cuff ensured that no light stimulation other than infrared light entered the sensing area. Slight adjustments were also made to the cuff pressure (20 to 25 mmHg) according to individual differences. The device was set for use on the third finger of the nondominant hand, with this finger kept at the same height as the heart. We confirmed the absence of any difference between use of the finger on the right or left hand for pulse detection.

An outline of the components for the FPG is shown in Figure 2B. The electric signal from the sensor was sent to a personal computer via a pressurized pulse wave-detecting device, and the digital signal was recorded at a sampling rate of 1 kHz for 1 s using Vital Vision version 4.03K software (CCI, Hakata, Japan). Frequency components with a lower limit of 0.5 Hz and an upper limit of 45 Hz were extracted using a band-pass filter; other nonspecific frequencies that would cause noise were attenuated.

### Indices of FPG

We obtained five indices from the FPG: finger pulse wave amplitude; chaotic attractor; the largest Lyapunov exponent; finger pulse rate; and autonomic nervous system indices. Periods for all data analysis were 3 min during pre- and post-task, and 3 min during each stage of the CWT. Sufficient data for analysis has previously been reported to be at least 2 min [29], so we extracted the most stable 3 min of data from the 10-min of the pre-task and post-task periods and from each stage of the CWT.

### Finger pulse wave amplitude

Finger pulse wave amplitude was defined as the mean difference between the lowest and highest points of observed data.

### Chaotic attractor constructed by nonlinear time-series analysis and the largest Lyapunov exponent

**Figure 3:**
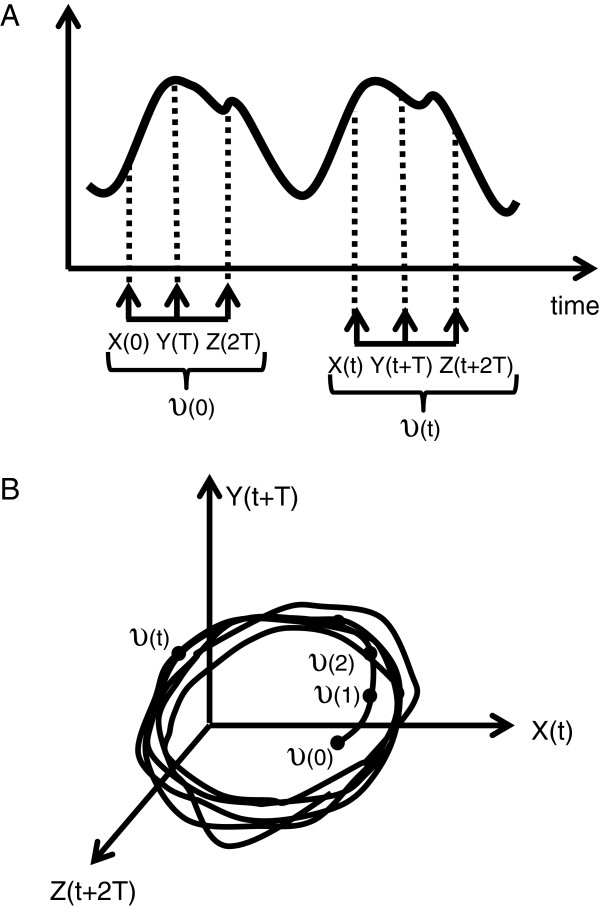
**Conceptual scheme of the reconstruction phase from observed nonlinear data using Taken’s embedding method. (A)***υ*(*t*) is the dimensional state vector, *X*, *Y* and *Z* represent original data, and *T* is the time delay. **(B)** Hypothetical reconstructed attractor in embedding *υ*(*t*) into three dimensions.

For the first step in nonlinear time analysis, as seen in Figure 3A, the phase space was reconstructed from FPG data based on Taken’s embedding method [30]. Phase space is an abstract mathematical space in which to view the chaos. A reconstructed phase can be described as follows:

$$\upsilon \left(t\right)=[X\left(t\right),Y\left(t+T\right),Z(t+\left(d-1\right)T]$$

where *υ*(*t*) is the dimensional state vector, *X*, *Y* and *Z* are original data, *d* is the number of embedding dimensions, and *T* is the time delay. An appropriate time delay *T* and embedding dimension *d* are important for reconstructing the attractor. According to a previous report [22], we determined default values (*T* = 50 ms; *d* = 3). Figure 3B presents a hypothetical reconstructed attractor in three dimensions. To visualize the state of peripheral dynamics, we constructed colorful images of the chaotic attractor. Since the attractor was derived from the finger pulse wave, the relative size of the attractor reflected the finger pulse wave amplitude. If the peripheral blood flow decreased, the size of the attractor decreased. The shape of the attractor was formed by the trajectory and showed the instability of the finger pulse wave, which was synchronized with the chaos status. If the level of chaos increased, the chaotic attractor became an awkward shape. The largest Lyapunov exponent, which quantitatively shows the level of chaos, was extracted from the attractor on the basis of the methods of Sano and Sawada [31]. An increase in the largest Lyapunov exponent signifies that the irregularity of the level of chaos has increased [32]. Generally, when healthy people have mental stress and try to overcome difficulties, the level of chaos will increase. Other indices of chaos in the human body were too complicated to examine in detail here, so we concentrated on the chaotic attractor and the largest Lyapunov exponent.

### Finger pulse rate and autonomic nervous system indices calculated by fast Fourier transform analysis

To obtain values for finger pulse rate, FPG was converted into accelerated plethysmography (APG), which is the second derivative of the FPG waveform. Because of the unstable baseline of FPG, Sano *et al*. [33] proposed using APG, which shows clear peaks, to facilitate analysis. Using the APG, we calculated autonomic nervous system indices by fast Fourier transform analysis [34]. A low-frequency (LF) component (0.04 to 0.15 Hz) and a high-frequency (HF) component (0.15 to 0.40 Hz) were extracted from the APG as indices of the autonomic nervous system activity. Like HRV, the HF component is an index of parasympathetic nerve activity, and the LF/HF ratio is an index of sympathetic nerve activity. Using a band-pass filter, other nonspecific frequencies, such as ultra-low frequencies, were excluded. To consider the effects of the menstrual cycle on autonomic nervous function, we divided participants into two phases of the menstrual cycle: the luteal phase (*n* = 16) and the follicular phase (*n* = 15) and compared those HF components and the LF/HF ratios.

### Statistical analysis

Measurement data used in statistical analyses were all tested with nonparametric tests. Values from each of the stages (pre-task, CWT-1, CWT-2, CWT-3, and post-task) were compared by calculating the respective means and standard deviations and applying the Wilcoxon signed-ranks test. The Spearman’s rank correlation coefficient rho was calculated to determine the correlation between indices. The Mann–Whitney U test was used to compare the autonomic nervous system indices of two phases of the menstrual cycle.

Analysis was performed using PASW Statistics for Windows version 20 statistical software (IBM Japan, Tokyo, Japan). *P* values < 0.05 obtained in 2-sided tests were considered statistically significant.

## Results

### Level of difficulty at each stage of CWT

Mean values for CWT score at each of the three stages of difficulty were: CWT-1, 82.7 ± 12.4%; CWT-2, 67.3 ± 17.4%; and CWT-3, 57.3 ± 20.3%. The overall mean was 67.5 ± 16.6%. A significant reduction in score was seen with increasing difficulty from CWT-1 to CWT-2 and from CWT-2 to CWT-3 (*P* < 0.001). The score was also significantly lower in CWT-3 than in CWT-1 (*p* < 0.001).

### Effects of mental stress on vital signs

Examination of vital signs for the 31 participants in each of the pre-task, CWT, and post-task periods showed that respiratory rate increased significantly from pre-task to CWT (*P* < 0.001) and decreased significantly from CWT to post-task (*P* < 0.001). Conversely, systolic blood pressure (SBP), diastolic blood pressure (DBP), and body temperature all showed no significant changes before and after the CWT.

### Effects of mental stress on psychological indices

**Figure 4:**
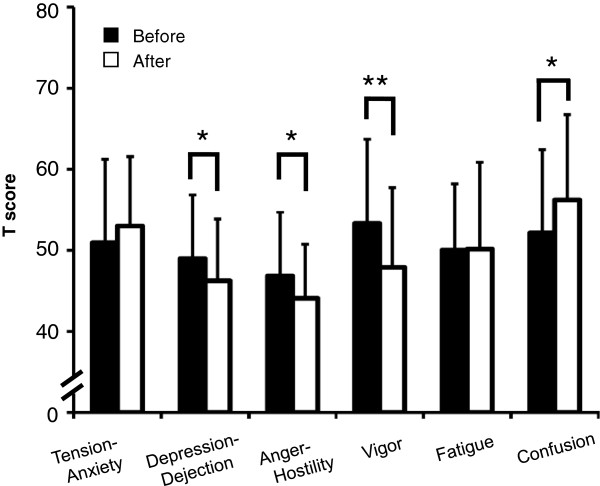
**Effect of Stroop color-word conflict test on mean standardized scores in POMS.** T scores for the six POMS scales were calculated, and scores before and after administration of the Stroop color-word conflict test were compared. Values represent mean ± standard deviation (*n* = 31). *P < 0.05; **P < 0.01 (Wilcoxon signed-ranks test).

Changes in psychological indices after CWT in each scale of the POMS (T score) are shown in Figure 4. After CWT, scores on the depression-dejection (*P* < 0.05), anger-hostility (*P* < 0.05), and vigor (*P* < 0.01) subscales were significantly decreased. Conversely, scores on the confusion subscale were significantly increased after CWT (*P* < 0.05). T score was calculated as follows:

$$\begin{array}{ll} T\;\mathrm{score}=50 & +10\\ & \times \left(\mathrm{raw}\;\mathrm{score}–\mathrm{mean}\;\mathrm{value}\right)/\text{standard deviation} \end{array}$$

### Effect of mental stress on FPG

**Figure 5:**
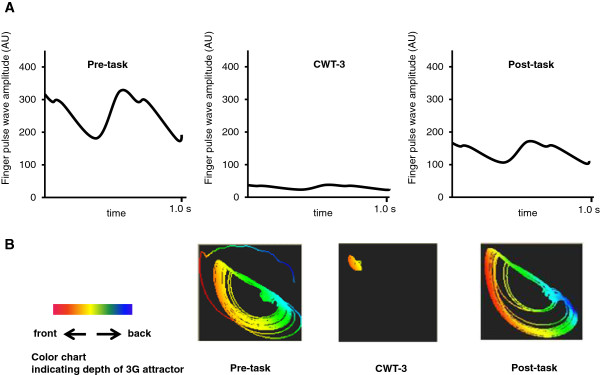
**Changes in finger pulse characteristics. (A)** Representative changes in finger pulse waveform. **(B)** Representative changes in chaotic attractor. Chaotic attractor was reconstructed by embedding nonlinear data of FPG into the dimensional phase. Differences in depth of the attractor are shown in color. AU, arbitrary unit; CWT, Color-word conflict test.

Changes in finger pulse rate after CWT are shown in Table 1. Finger pulse rate during CWT was significantly increased compared with both pre-task (*P* < 0.001) and post-task (*P* < 0.001). Changes in the largest Lyapunov exponent and finger pulse wave amplitude in each stage are shown in Table 2. There were no significant changes in the largest Lyapunov exponent. In contrast, finger pulse wave amplitude was significantly decreased during CWT (*P* < 0.001). Figure 5A shows representative changes in the finger pulse waveform and amplitude. In particular, the waveform became almost flat and pulse wave amplitude was considerably decreased during CWT-3. During CWT-3, the chaotic attractor shrank and became rigid (Figure 5B). Although we could not observe the exact shape of the attractor, the depth appeared shallow.

**Table 1:** Changes in finger pulse rate

**Finger pulse rate (bpm)**	
**Pre-task**	67.13 ± 10.38
**CWT-1**	75.39 ± 12.47
**CWT-2**	75.32 ± 11.94
**CWT-3**	76.52 ± 11.78
**Post-task**	68.13 ± 10.44

Values are given as mean ± standard deviation (*n* = 31). CWT, Stroop color-word conflict test; post-task, rest period after CWT; pre-task, rest period before CWT. Significant differences (*P* < 0.001) were seen in all stages. (Wilcoxon signed-ranks test).

**Table 2:** Mean values of largest Lyapunov exponent and finger pulse wave amplitude

	**Lyapunov exponent (AU)**	**Finger pulse wave amplitude (AU)**
**Pre-task**	3.34 ± 0.75	210 ± 109
**CWT-1**	3.83 ± 1.36	93 ± 89
**CWT-2**	3.63 ± 1.31	103 ± 91
**CWT-3**	3.66 ± 1.36	109 ± 89
**Post-task**	3.61 ± 1.05	174 ± 87

Values are given as mean ± standard deviation (*n* = 31). AU, arbitrary unit; CWT, Stroop color-word conflict test; post-task, rest period after CWT; pre-task, rest period before CWT. Significant differences (*P* < 0.001) were seen in all stages of finger pulse wave amplitude. (Wilcoxon signed-ranks test).

### Effect of mental stress on autonomic nervous system

Changes in the HF component and the LF/HF ratio, which are known as indices of the autonomic nervous system activity, were examined by comparing values derived from APG for all pre-task, CWT, and post-task conditions (Table 3). The HF component significantly decreased (*P* < 0.001) and the LF/HF ratio significantly increased (*P* < 0.01) during the CWT. As for the effect of the menstrual cycle on the autonomic nervous system, the HF component in the follicular phase was significantly higher than in the luteal phase during pre-task and CWT-2 (*P* < .05), but other indices were similar in both phases. An additional table file shows this in more detail (see Additional file 1: Table S1).

**Table 3:** Mean values of autonomic nervous system indices derived from APG

	**HF (ms^2^)**	**LF/HF**
**Pre-task**	5.97 ± 0.70	1.03 ± 0.15
**CWT-1**	5.25 ± 0.91	1.06 ± 0.20
**CWT-2**	5.11 ± 0.82	1.12 ± 0.20
**CWT-3**	4.95 ± 0.98	1.12 ± 0.30
**Post-task**	5.88 ± 0.59	1.07 ± 0.13

Values are given as mean ± standard deviation (*n* = 31). APG, acceleration plethysmography; CWT, Stroop color-word conflict test; HF, High-frequency component; LF, low-frequency component; post-task, rest period after CWT; pre-task, rest period before CWT. Significant differences were seen in all stages of HF (*P* < 0.001) and between Pre-task and Post-task of LF/HF (*P* < 0.05). (Wilcoxon signed-ranks test).

### Correlations between POMS and physiological indices

Correlation coefficients rho between POMS and the HF component, the LF/HF ratio, finger pulse rate, finger pulse wave amplitude, and the largest Lyapunov exponent are shown in Table 4. There are some negative correlations: Post-task scores on the tension-anxiety subscale correlated negatively with the largest Lyapunov exponent during CWT-1 (*P* < 0.01), and with the LF/HF ratio during CWT-3 (*P* < 0.05), and post-task scores on the fatigue subscale correlated negatively with the LF/HF ratio during CWT-2 (*P* < 0.01).

**Table 4:** Correlations between POMS and physiological values in each condition

	**POMS pre-task**	**Tension-anxiety**	**Depression-dejection**	**Anger-hostility**	**Vigor**	**Fatigue**	**Confusion**
**Pre-task**	HF	0.17	0.10	-0.04	-0.10	0.19	-0.02
	LF/HF	0.16	0.13	0.18	-0.02	0.06	-0.02
	Finger pulse rate	0.11	0.05	0.09	-0.13	-0.27	0.01
	Pulse amplitude	-0.17	-0.14	-0.15	0.03	0.10	-0.01
	Lyapunov exponent	0.01	-0.26	-0.15	-0.06	-0.15	0.08
	**POMS post-task**	**Tension-anxiety**	**Depression-dejection**	**Anger-hostility**	**Vigor**	**Fatigue**	**Confusion**
**CWT-1**	HF	-0.13	-0.27	0.10	-0.27	-0.02	-0.09
	LF/HF	-0.14	-0.03	-0.06	0.22	-0.17	-0.07
	Finger pulse rate	-0.71	0.21	-0.22	-0.06	0.01	-0.05
	Pulse amplitude	-0.09	-0.21	0.09	-0.21	-0.17	0.06
	Lyapunov exponent	-**0**.47^**^	-0.25	-0.05	0.15	-**0**.**31**	-0.28
**CWT-2**	HF	-0.24	0.03	-0.03	-0.24	0.14	-0.13
	LF/HF	-0.29	-0.29	-0.05	0.06	-**0**.46^**^	-0.14
	Finger pulse rate	-0.05	0.20	-0.09	-0.15	-0.01	-0.07
	Pulse amplitude	-0.08	-0.26	0.20	-0.22	-0.17	0.11
	Lyapunov exponent	-**0**.**34**	-0.13	0.02	0.05	-0.21	-0.28
**CWT-3**	HF	-0.29	0.12	0.07	-**0**.**35**	-0.07	-0.06
	LF/HF	-**0**.36^*^	-**0**.**32**	-0.05	0.10	-**0**.**33**	-**0**.**32**
	Finger pulse rate	-0.03	0.04	-0.14	-0.15	-0.06	-0.02
	Pulse amplitude	0.01	-0.14	0.16	-0.23	-0.13	0.22
	Lyapunov exponent	-**0**.**31**	-0.11	0.07	-0.09	-0.25	-0.20
**Post-task**	HF	-0.12	0.06	-0.01	0.09	0.04	-0.09
	LF/HF	-0.09	-0.21	0.26	-0.23	-**0**.**33**	-0.06
	Finger pulse rate	0.08	0.14	-0.02	-0.13	-0.07	-0.05
	Pulse amplitude	-0.21	-0.06	-0.08	0.04	-0.17	-0.01
	Lyapunov exponent	-0.18	-0.14	0.09	-0.04	-0.02	-0.12

Values are given as Spearman’s rank correlation coefficient rho (*n* = 31). Values in bold had >0.30 correlation with POMS. *P < 0.05; **P < 0.01 (Spearman’s rank correlation). CWT, Stroop color-word conflict test; HF, high-frequency component; LF, low-frequency component; post-task, rest period after CWT; pre-task, rest period before CWT.

## Discussion

Our results demonstrate that physical changes induced by mental stress can be observed by indices derived from FPG. Therefore, FPG can be one of the easiest methods by which to evaluate mental stress quantitatively. In particular, the largest Lyapunov exponent and the LF/HF ratio might be associated with acute mental change.

### Validity of CWT as stress-inducing test

The CWT is known as a psychological stress test; several reports have described increased pulse rate and inhibited parasympathetic autonomic nerve activity during CWT [27,35,36]. As shown in Table 1, participants in this study showed significantly increased finger pulse rate during the test, by comparison with the rest periods. We also set three stages of difficulty for each task. In practice, a stepwise reduction in the correct response rate was found, affirming the gradual increase in difficulty. The values indicate that the finger pulse rate during testing was always higher than during the rest periods. Compared with other indices, SBP, DBP, and body temperature did not change after CWT. Finger pulse rate, respiratory rate, the LF/HF ratio, and the HF component were measured during CWT, but SBP and DBP were measured after not measure the blood pressure during CWT because the left arm was used for the measurement of FPG and the right hand was used for clicking the mouse. Although we conjectured that these were changed during CWT as well as other indices, we could not determine the change exactly in the present study. To overcome this problem, we should instead use a stress test in which a participant’s hand is not used.

The POMS also showed decreased ratings in the vigor subscale and increased ratings in the confusion subscale immediately after the test (Figure 4). Ratings in the depression-dejection and anger-hostility subscales may have decreased significantly after CWT as a side effect of the concentration workload. These results indicate that the CWT in this study was sufficient to cause acute confusion and reduce vigor. Although ratings in the tension-anxiety and fatigue subscales showed no significant changes, correlations between them and some physiological indices showed significant changes (Table 3). Physiological indices might reflect a slight mental change, but this point is unclear in this short-term study. In future studies, we should improve the mental test (for example, we could lengthen the periods of the task-time).

### Acute mental stress affects peripheral circulation

Generally, sympathetic nerve activity is parallel to parasympathetic nerve activity. In our results, the HF component was decreased and the LF/HF ratio was increased during CWT, showing that mental stress activated sympathetic nerve activity and inhibited parasympathetic nerve activity. We speculate that during mental workload, peripheral circulation decreases with activation of sympathetic nerve balance. The FPG reportedly reflects sympathetic nerve activity [37], and alpha-adrenergic sympathetic nerve activity in the skin of the fingertips increases as a result of psychological stimulation [6,38]. Increased sympathetic nerve activity due to psychological and physical strain is commonly known to cause constriction of peripheral arteries. This vasoconstriction reduces blood flow and subsequently leads to decreased amplitude of the finger pulse wave [39,40]. Based on these reports, we believe that the finger pulse wave amplitude decreased due to increased peripheral sympathetic nerve activity.

### Evaluation of mental stress by chaotic indices

In this study, the finger pulse wave amplitude decreased and the chaotic attractor shrank during CWT (Table 2 and Figure 5B). The results indicate that the decrease in peripheral circulation was induced by mental stress. Tsuda *et al*. [22] demonstrated that orbital unevenness of the chaotic attractor became small under treatment, and the attractors of patients with low ratings for all scores showed stiffening. Previous studies suggested that mental stress decreased peripheral circulation and dynamics [20,41]. Since significant changes to the first Lyapunov exponent were not found in our study, we could not reveal any changes in peripheral dynamics. Moreover, we could not determine the clear shape of the chaotic attractor during testing. We therefore should improve the system of chaos analysis to show a clear shape even when the size of the attractor is small. It is difficult to determine the exact chaotic state, because of dependence on several physical and mental conditions, so more experiments are needed to investigate dynamics in the human body from different perspectives.

### Relation between physiological indices and POMS

It seems that the LF/HF ratio tends to reflect scores on the fatigue subscale sensitively, whereas the largest Lyapunov exponent tends to reflect scores on the tension-anxiety subscale (Table 4). Interestingly, there were no correlations between them during rest periods, but strong correlations appeared during CWT. This result suggests that the LF/HF ratio and the largest Lyapunov exponent could be valuable indices to reflect emotional changes instantly. Imanishi *et al*. [23] reported that the largest Lyapunov exponent had positive correlations with anxiety and fear. They also found no relationship between heart rate and emotions. Because they used task-induced feelings of anxiety, fear and relief, our results did not coincide with their results. Correlations between the Lyapunov exponent and mental stress may change depending on the situation. Further investigations are needed to confirm the association between other emotional states and physiological indices. One difficulty in testing accurate correlations between subjective and objective data was the difference in timing of the measurements. The POMS questionnaire was completed before the rest period and after the task, whereas hand psychological indices were measured during the rest period and CWT. Although we are not able to measure both types of data at the same time, they should be measured simultaneously as much as possible to overcome this problem.

### Effect of menstrual cycle on autonomic nervous system

The follicular phase is the period when concentration of estrogen is gradually increasing. Previous studies had indicated that parasympathetic activity presented by the HF component was influenced by estrogen [42]. Thus, we conjectured that a higher level of estrogen was observed in the follicular phase than in the luteal phase during the pre-task, CWT-1 and CWT-2. However, such differences disappeared in the CWT-3 and post-task periods. This may have been because the strong mental stress might cause the large individual differences in these periods. We simply divided the menstrual cycle into two phases depending on participants’ responses. Future investigations should consider monitoring hormone levels and measuring the basal body temperatures before the experiment to more reliably identify the menstrual phases.

### Clinical implications of FPG

Since FPG is noninvasive, the device can be made portable with simple modifications, and measurements can be performed anywhere. For example, desktop measurements could be performed in the workplace or at the hospital bedside in patients who are unable to express feelings, thus facilitating the identification of mental stress. Fujimoto and Yamaguchi [19] suggested that the chaotic attractor could be used to monitor the vital condition and support older people. To add to their idea, we suggest that it is better to calculate not only the chaotic attractor but also other indices, such as the autonomic nervous system activity and the largest Lyapunov exponent. Moreover, it can be used for self-checks by workers who perform heavy labor to assess their own stress levels visually and increase the awareness of stress-related disease prevention. Since FPG provides an estimate of artery stiffness [43], it is currently used for indices of blood vessel age [44]. The measurement environment is still limited because of its sharp sensitivity, but indices derived from FPG may reflect not only the blood vessel status, but also mental stress.

Chaos indices such as the chaotic attractor have not been used to date in the medical field, because of the complex nature of the calculations involved. However, improvements in analytical techniques due to new computer technology have facilitated the use of chaos indices, which are expected to be developed for clinical application. If chaos indices are analyzed in greater detail with relation to other factors, such as clinical condition or lifestyle habits, larger amounts of health-related information can easily be acquired. One study noted that analyzing nonlinear characteristics plays a role in predicting some illnesses of the cardiovascular system [45]. Further studies aimed at achieving clinical applications are necessary to investigate not only how chaos indices change in response to stress, but also to emotional patterns of restlessness, anger, and happiness.

### Specific comments: study limitations

Our sample size was small and only comprised young, healthy women, so the results may not be generalizable to other populations (for example, men or older individuals). Furthermore, CWT is a validated and widely used test for inducing stress, but is not the same as stress in real life, of which chronically high levels are associated with negative effects on health. Thus, it is necessary to examine the use of FPG in more real-life situations.

In addition, the indices derived from FPG are strongly dependent on peripheral circulation, so we cannot use it for patients who have cardiovascular disease. We also cannot use it when the peripheral vessel is too contracted to measure.

## Conclusions

The indices of autonomic nervous system activity and other physical indices derived from FPG were changed by mental stress. The diminished peripheral blood flow caused by mental stress during testing was observed by a reduction of the finger pulse wave amplitude and shrinkage of chaotic attractor. The largest Lyapunov exponent and the LF/HF ratio might be associated with the tension-anxiety and fatigue subscales on the POMS, respectively. Our findings indicate that FPG is one of the easiest methods to evaluate mental stress quantitatively.

## Abbreviations

APG: Acceleration plethysmography; CWT: Stroop color-word conflict test; DBP: Diastolic blood pressure; FPG: Finger plethysmography; HF: High-frequency component; HRV: Heart rate variability; LF: Low-frequency component; POMS: Profile of mood states; SBP: Systolic blood pressure.

## Competing interests

The authors declare that they have no competing interests.

## Authors’ contributions

EM designed and carried out the study, performed the statistical analysis and wrote the manuscript. EO was engaged in the drafting of the study design, helped with the data measurement of the subjects, the analysis of psychology data and correction of the manuscript. JO helped with the drafting of the study design, assistance to data measurement and the interpretation of data. SS, MH and MM helped with the data measurement of the subjects. JH helped with programming to make Stroop color-word conflict test. Administrative supervision was provided by KM and TK. All authors read and approved the final manuscript.

## Supplementary Material

Additional file 1: Table S1 Mann–Whitney U test for comparing physiological indices of two phases of the menstrual cycle. We conducted the Mann–Whitney U test to show differences of physiological indices between two phases of the menstrual cycle. Significant differences were only shown in the HF component during pre-task and CWT-2 (*P* < 0.05). Other indices did not show any differences by the menstrual cycle. The menstrual cycle might influence the HF component under the rest-condition, but strong mental stress might cause the large individual differences. *n* = 31 (luteal phase = 16, follicular phase = 15). CWT, Stroop color-word conflict test; post-task: rest period after CWT; pre-task: rest period before CWT. *P < 0.05. Click here for file
